# A LQR-Based Controller with Estimation of Road Bank for Improving Vehicle Lateral and Rollover Stability via Active Suspension

**DOI:** 10.3390/s17102318

**Published:** 2017-10-13

**Authors:** Andres Riofrio, Susana Sanz, Maria Jesus L. Boada, Beatriz L. Boada

**Affiliations:** Mechanical Engineering Department, Institute for Automotive Vehicle Safety (ISVA), Universidad Carlos III de Madrid, Avda. de la Universidad 30, 28911 Madrid, Spain; ariofrio@ing.uc3m.es (A.R.); ssanz@ing.uc3m.es (S.S.); mjboada@ing.uc3m.es (M.J.L.B.); bboada@ing.uc3m.es (B.L.B.)

**Keywords:** bank, LQR, active suspension, Kalman filter, roll angle, yaw rate, sideslip, load transfer

## Abstract

In this article, a Linear Quadratic Regulator (LQR) lateral stability and rollover controller has been developed including as the main novelty taking into account the road bank angle and using exclusively active suspension for both lateral stability and rollover control. The main problem regarding the road bank is that it cannot be measured by means of on-board sensors. The solution proposed in this article is performing an estimation of this variable using a Kalman filter. In this way, it is possible to distinguish between the road disturbance component and the vehicle’s roll angle. The controller’s effectiveness has been tested by means of simulations carried out in TruckSim, using an experimentally-validated vehicle model. Lateral load transfer, roll angle, yaw rate and sideslip angle have been analyzed in order to quantify the improvements achieved on the behavior of the vehicle. For that purpose, these variables have been compared with the results obtained from both a vehicle that uses passive suspension and a vehicle using a fuzzy logic controller.

## 1. Introduction

Rollover in heavy duty vehicles is one of the kinds of accidents with worse consequences for passengers. Even though the low percentage of accidents in which this risk circumstance is achieved, 2.1% according to the National Highway Traffic Safety Administration (NHTSA), in 2010, 35% of the total of fatal vehicle accidents involved rollover [1]. These data have not improved in recent years. In 2015, 7210 passenger vehicle occupants died in rollover crashes in the U.S. out of 22,543 deaths considering all types of accidents [2]. These numbers show the importance of rollover prevention devices. Previous works [3,4,5] show rollover controllers based on different methods to achieve the desired behavior of the vehicle, such as differential braking, four-wheel steering or active stabilizers. Anti-roll bars were used as actuators in [6], where a controller was developed by means of neural networks to improve roll stability. In [7,8], a Unified Chassis Control (UCC) system is designed to prevent rollover using a rollover index and a model-based roll state estimator. These systems have a two-level control structure, including actuation on both suspension and the steering and braking systems. Other authors have designed systems to control lateral stability, as well as roll dynamics. In [9], an integrated chassis control to improve lateral stability was developed using Active Four-Wheel Steering (AFWS). In [10], a strategy based on the estimated sideslip is proposed for stability control. In [11], a lateral dynamics controller was designed using Direct Control over Yaw moment (DYC) and a two degrees of freedom model. A lateral stability controller was developed in [12] using active steering (AFS) and DYC by means of a gain scheduling approach. Motor driving and regenerative braking torque distribution control were used in [13] to improve the vehicle’s stability. In [14], a lateral and rollover stability controller was designed that mitigates the effect of delay in active braking actuation systems. These methodologies are easy to implement in production vehicles given that these devices are already installed in vehicles.

One major drawback of the mentioned approaches is that the main function of braking or steering systems is not to prevent rollover. This is why active suspension can play an important role in this issue as its primary function is to ensure roll stability. In [15], a Takagi–Sugeno fuzzy control system for semi-active suspension was developed for a quarter vehicle model, but no influence on roll or lateral dynamics of the vehicle was studied.

On the other hand, previous research has established that the road bank angle affects directly both lateral and roll dynamics of the vehicle [16]. In [16], it is also stated that road bank estimation allows preventing a false activation of the controller, as disturbances on the road may show up as a possible rollover situation for those systems that are not taking into account this variable.

The main problem regarding the road bank is that this variable cannot be measured directly by means of on-board sensors, being the biggest problem for its estimation to distinguish between the road disturbance component and the vehicle’s rollover using typical roll measurements [17].

There exist several works in which the estimation of the sideslip angle for lateral stability control has been performed by means of the estimated roll angle [18,19], but the roll angle and road bank were not estimated independently.

Estimation of the road bank was performed in [20] by means of a Dynamic Simplex Algorithm (DSA), even though a controller was not developed, but rather an early warning system. In [21], the road bank and roll angle were estimated by means of an unknown input sliding mode. In [22], the road bank was estimated using a constrained dual Kalman filter.

The main novelty of this paper is that a combined method for the estimation of the road bank and rollover control has been designed. In previous works, both rollover control and estimators for the road bank angle have been found, but in none were both methodologies applied at the same time. In addition, an adaptation of the continuous Linear Quadratic Regulator (LQR) has been performed, so that perturbations and non-zero reference state values could be taken into account. An LQR controller has been used as it offers great robustness, at the same time offering a simple solution to a complex non-linear problem. The proposed controller acts only on an active-suspension in order to improve both roll and lateral stability. The majority of previous works act on braking system, additionally, to perform this same task.

The behavior of the vehicle has been simulated by means of TruckSim, using a validated model of a Mercedes Benz Sprinter van.

This article is organized as follows. In Section 2, the equations describing the vehicle model are stated. In Section 3, the state equations used for the estimation of state variables are developed, including the estimation of the road bank. In addition, a brief theoretical explanation of the Kalman filter is depicted. Section 4 is focused on defining the state equations used for the controller and the calculation of the control vector. In Section 5, the proposed controller architecture is developed, as well as the experimental validation for the simulation model is shown. A quantitative comparison between the effectiveness of different controllers and the proposed system in different environments is shown in Section 6, including lateral load transfer, roll angle, yaw rate and sideslip angle.

## 2. Vehicle Model

**Figure 1:**
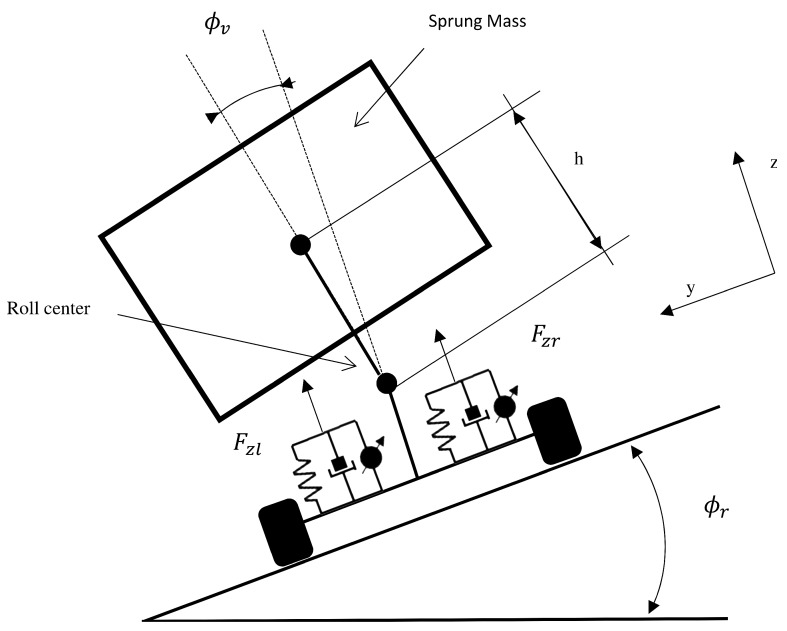
Flat roll model.

**Figure 2:**
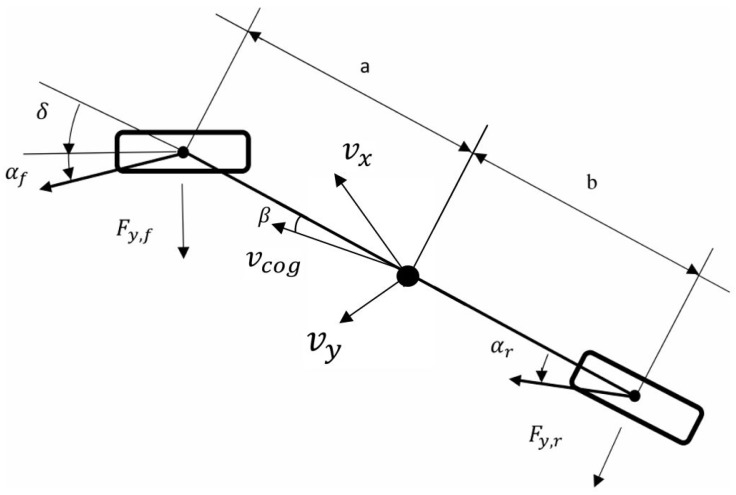
Bike lateral motion model.

In order to be able to develop a system that controls both lateral and roll dynamics of the vehicle taking into account the effect of the road bank, it is necessary to use a vehicle model that allows identifying vehicle roll angle and road bank as separate variables. A flat vehicle model that considers the roll dynamics (see Figure 1) is used in this paper [17]. The single track model is used to represent the lateral dynamics of the vehicle, assuming that the difference between the slip angles of the inner and outer wheels is small (see Figure 2).

Taking into account the assumptions described in [17], the equations for motion are as follows:

(1) $$\overset{\cdot }{\beta }=-\frac{I_{eq}C_{0}}{I_{x}mv_{x}}\beta -\left(1+\frac{I_{eq}C_{1}}{I_{x}mv_{x}^{2}}\right)r_{f}+\frac{h(mgh-k_{r})}{I_{x}v_{x}}\phi_{v}-\frac{hb}{I_{x}v_{x}}{\overset{\cdot }{\phi }}_{v}+\frac{I_{eq}C_{\alpha f}}{I_{x}mv_{x}}\delta -\frac{g}{v_{x}}{\overset{\cdot }{\phi }}_{r}$$

(2) $${\overset{\cdot }{r}}_{f}=\frac{C_{1}}{I_{z}}\beta -\frac{C_{2}}{I_{z}v_{x}}r_{f}+\frac{aC_{\alpha f}}{I_{z}}\delta$$

(3) $${\overset{\cdot \cdot }{\phi }}_{v}=\frac{C_{0}h}{I_{x}}\beta -\frac{C_{1}h}{I_{x}v_{x}}r_{f}+\frac{mgh-k_{r}}{I_{x}}\phi_{v}-\frac{b_{r}}{I_{x}}{\overset{\cdot }{\phi }}_{r}+\frac{C_{\alpha f}h}{I_{x}}\delta -{\overset{\cdot }{p}}_{f}+\frac{t_{r}F_{zl}}{2I_{x}}{\overset{\cdot }{\phi }}_{v}-\frac{t_{r}F_{zl}}{2I_{x}}{\overset{\cdot }{\phi }}_{v}$$

where,

(4) $$C_{0}{=C}_{\alpha f}+C_{\alpha r}$$

(5) $$C_{1}{=aC}_{\alpha f}-{bC}_{\alpha r}$$

(6) $$C_{2}{=a^{2}C}_{\alpha f}+{b^{2}C}_{\alpha r}$$

(7) $$I_{eq}{=I}_{x}+mh^{2}$$

## 3. Kalman Filter Estimation

For this paper, a Linear Kalman Filter (LKF) was implemented in order to estimate all the vehicle states, including those that cannot be directly measured using sensors.

To perform this estimation, the equations shown in Section 2 were written into state equations by adding the corresponding terms to the vertical forces exerted by the active suspension system, **u${}_{e}$** being the input vector for the model:

(8) $${\overset{\cdot }{x}}_{e}=A_{e}x_{e}+B_{e}u_{e}$$

(9) $$y={\mathrm{Hx}}_{e}$$

where:

$$x_{e}={\left[\begin{array}{cccccccc} \beta & r_{f} & \phi_{v} & {\overset{\cdot }{\phi }}_{v} & \phi_{r} & P_{f} & {\overset{\cdot }{p}}_{f} & \varepsilon_{r} \end{array}\right]}^{T}$$

$$A_{e}=\left[\begin{array}{cc} A & B_{w}\\ 0 & A_{w} \end{array}\right]$$

$$A=\left[\begin{array}{cccc} -\frac{I_{eq}C_{0}}{I_{x}mv_{x}} & -\left(1+\frac{I_{eq}C_{1}}{I_{x}mv_{x}^{2}}\right) & \frac{h(mgh-k_{r})}{I_{x}v_{x}} & -\frac{hb}{I_{x}v_{x}}\\ -\frac{C_{1}}{I_{z}} & -\frac{C_{2}}{I_{z}v_{x}} & 0 & 0\\ 0 & 0 & 0 & 1\\ \frac{C_{0}h}{I_{x}} & -\frac{C_{1}h}{I_{x}v_{x}} & \frac{mgh-k_{r}}{I_{x}} & -\frac{b_{r}}{I_{x}} \end{array}\right]$$

$$B_{w}=\left[\begin{array}{cccc} \frac{-g}{v_{x}} & 0 & 0 & 0\\ 0 & 0 & 0 & 0\\ 0 & 0 & 0 & 0\\ 0 & 0 & -1 & 0 \end{array}\right]$$

$$A_{w}=\left[\begin{array}{cccc} 0 & 1 & 0 & 1\\ 0 & 0 & 1 & 0\\ 0 & 0 & 0 & 0\\ 0 & 0 & 0 & 0 \end{array}\right]$$

$$B_{e}=\left[\begin{array}{ccc} \frac{I_{eq}C_{\alpha f}}{I_{x}mv_{x}} & 0 & 0\\ \frac{aC_{\alpha f}}{I_{z}} & 0 & 0\\ 0 & 0 & 0\\ \frac{C_{\alpha f}h}{I_{x}} & 0 & 0\\ 0 & \frac{t_{r}}{2I_{x}} & -\frac{t_{r}}{2I_{x}}\\ 0 & 0 & 0\\ 0 & 0 & 0\\ 0 & 0 & 0 \end{array}\right]$$

$$u_{e}={\left\{\begin{array}{ccc} \delta & F_{zl} & F_{zr} \end{array}\right\}}^{T}$$

$$H=\left[\begin{array}{cccccccc} 1 & 0 & 0 & 0 & 0 & 0 & 0 & 0\\ 0 & 1 & 0 & 0 & 0 & 0 & 0 & 0\\ 0 & 0 & 1 & 0 & 1 & 0 & 0 & 0\\ 0 & 0 & 0 & 1 & 0 & 1 & 0 & 0 \end{array}\right]$$

and assuming a small pitch angle of the vehicle [17]:

$${\dot{\phi }}_{r}\approx P_{f}+\varepsilon_{r}$$

where:

$$P_{f}\approx 0$$

$$\varepsilon_{r}\approx 0$$

The steering wheel angle and the vertical forces exerted by the active suspension system were considered as inputs for the system **u${}_{e}$**. In this case, the estimated variables in this case were the sideslip angle, yaw rate, roll rate and the road bank angle.

To simulate the behavior of the controller installed on a real vehicle where sensor data are obtained in each sample time, the discrete state space system had to be formulated. The discrete space system was defined using the first order approximation of Euler:

(10) $$\dot{x}=\frac{x_{k}-x_{k-1}}{T_{S}}$$

where $T_{s}$ is the sampling time. Assuming that the system in Equation (8) is linear, according to [23], the discrete system can be expressed as:

(11) $$x_{k+1}=A_{\mathrm{de}}x_{k}+B_{\mathrm{de}}u+w_{k}$$

and:

(12) $$y_{k}={\mathrm{Hx}}_{k}+v_{k}$$

where:

- **A${}_{\mathrm{de}}$** is the discrete matrix **A** for estimation and is calculated as follows, with $T_{s}=0.001\;s$:
  (13) $$A_{\mathrm{de}}=\left(I+T_{s}A_{e}\right)$$
- **B${}_{\mathrm{de}}$** is the discrete matrix **B** for estimation, which is equal to **B${}_{e}$**$T_{s}$.
- **x${}_{k}$** represents the state vector for instance k.
- **w${}_{k}$** is the process noise vector for instance k, calculated assuming the normal Gaussian normal distribution as:
  $$w_{k}=N\left(0,Q_{e}\right)$$
- **v${}_{k}$** is the output noise vector for instance k, calculated assuming the normal Gaussian normal distribution as:
  $$v_{k}=N\left(0,R_{e}\right)$$
- **y${}_{k}$** is the output vector for instance k.

The linear Kalman filter, as shown in detail in [23], can be summarized as follows:

(1) The prediction of the state is given by:
  (14) $${\tilde{x}}_{k|k-1}=A_{\mathrm{de}}{\tilde{x}}_{k-1|k-1}+B_{\mathrm{de}}u_{e}k$$
(2) The predicted error covariance is calculated by means of:
  (15) $$P_{k|k-1}=A_{\mathrm{de}}P_{k-1|k-1}A_{\mathrm{de}}^{T}+Q_{e}$$
  where:
  $$Q_{e}=1e^{-2}\cdot I_{8x8}$$
(3) Therefore, the Kalman Gain is:
  (16) $$K_{k}=P_{k|k-1}+H^{T}{\left[{\mathrm{HP}}_{k-1|k-1}H^{T}+R_{e}\right]}^{-1}$$
  where:
  $$R_{e}=1e^{-4}\cdot I_{4x4}$$
(4) The state estimation is updated with measurement $y_{k}{,}_{m}$:
  (17) $${\tilde{x}}_{k|k}={\tilde{x}}_{k|k-1}+K_{k}\left[y_{k,m}-H{\tilde{x}}_{k|k-1}\right]$$
(5) The error covariance is updated:
  (18) $$P_{k|k}=\left[I-K_{k}H\right]P_{k|k-1}$$

with **x**${}_{0|0}$:

$$x_{k|k}={\left[\begin{array}{cccccccc} 0 & 0 & 0 & 0 & 0 & 0 & 0 & 0 \end{array}\right]}^{T}$$

and **P**${}_{0|0}$:

$$P_{k|k}=\left[\begin{array}{cccccccc} 1^{2} & 0 & 0 & 0 & 0 & 0 & 0 & 0\\ 0 & 0.1^{2} & 0 & 0 & 0 & 0 & 0 & 0\\ 0 & 0 & 0.01^{2} & 0 & 0 & 0 & 0 & 0\\ 0 & 0 & 0 & 0.01^{2} & 0 & 0 & 0 & 0\\ 0 & 0 & 0 & 0 & 0.1^{2} & 0 & 0 & 0\\ 0 & 0 & 0 & 0 & 0 & 0.1^{2} & 0 & 0\\ 0 & 0 & 0 & 0 & 0 & 0 & 0.1^{2} & 0\\ 0 & 0 & 0 & 0 & 0 & 0 & 0 & 0.1^{2} \end{array}\right]$$

The initial state value, **x**${}_{0|0}$, was the same one used by the TruckSim software to begin the simulations. For each step k, the inputs to the Kalman filter were updated with the data imported to Simulink from TruckSim, performing the state estimation for step k as an output of the Kalman filter.

The Kalman filter module was programmed in Simulink so that the iteration started and stopped when the vehicle ended the maneuver or the vehicle rolled over. The initial and final times were defined in TruckSim, and they are sent to Simulink at the beginning of the simulation.

## 4. LQR Controller

The determination of the optimal actuation of the vehicle’s active suspension system was not developed by means of an LQR controller. The model used in Section 3 was not valid for this implementation given that Equation (8) was not controllable. For this reason, the model was rewritten as shown in Equation (19). In this new model, two inputs were considered; the steering wheel angle and the road bank angle; and two control variables, the vertical exerted forces:

(19) $$x_{c,k+1}=A_{dc}x_{c,k}+B_{dc}v_{k}+C_{dc}u_{c,k}$$

where:

- **A${}_{\mathrm{dc}}$** is the discrete A matrix for control, calculated from matrix *A* in Section 3 as:
  (20) $$A_{\mathrm{dc}}=\left(I+T_{s}A\right)$$
- **v**${}_{k}$ is the input vector:
  $$v_{k}={\left[\begin{array}{cc} \delta & \phi_{r} \end{array}\right]}^{T}$$
- **u${}_{c,k}$** is the control vector:
  $$u_{c,k}={\left[\begin{array}{cc} F_{zl} & F_{zr} \end{array}\right]}^{T}$$

and:

$$x_{c,k}={\left[\begin{array}{cccc} \beta & r_{f} & \phi_{v} & \overset{\cdot }{\phi_{v}} \end{array}\right]}^{T}$$

$$B_{dc}=\left[\begin{array}{cc} \frac{I_{eq}C_{\alpha f}}{I_{x}mv_{x}} & \frac{-g}{v_{x}}\\ \frac{aC_{\alpha f}}{I_{z}} & 0\\ 0 & 0\\ \frac{C_{\alpha f}h}{I_{x}} & 0 \end{array}\right]T_{s}$$

$$C_{dc}=\left[\begin{array}{cc} 0 & 0\\ 0 & 0\\ 0 & 0\\ \frac{t_{r}}{2I_{x}} & \frac{-t_{r}}{2I_{x}} \end{array}\right]T_{s}$$

To control the vehicle’s roll and lateral stability, the LQR theory defined the performance index as follows [24]:

(21) $$J=\sum_{k=0}^{N-1}\left[w_{1}{\left(\beta_{k}-\beta_{d,k}\right)}^{2}+w_{2}{\left(r_{f,k}-r_{fd.k}\right)}^{2}+w_{3}{\left(\phi_{k}-\phi_{d,k}\right)}^{2}+w_{4}{\left({\dot{\phi }}_{k}-{\dot{\phi }}_{d,k}\right)}^{2}+w_{5}M_{x,k}\right]$$

where:

- $w_{i}$, with i = 1–5, are the factors indicating the influence of each variable;
- The subindex *d* indicates the desired value for that state variable;
- $M_{x}$ is the moment performed around the *x* axis to control the vehicle, which can be expressed as:
  (22) $$M_{x}={\mathrm{KDu}}_{c}$$
  with:
  D=trt22−trt22T

Equation (21) can be rewritten into the standard optimal control expression for the discrete problem as shown in Equation (23) [24]:

(23) $$J=\frac{1}{2}\sum_{k=0}^{N-1}\left(u_{c,k}^{T}{\mathrm{Ru}}_{c,k}\right)+{\left(x_{d,k}-x_{k}\right)}^{T}Q\left(x_{d,k}-x_{k}\right)$$

where:

- **x${}_{d,k}$** is the desired response of the state vector in sample *k*:
  $$x_{d,k}={\left[\begin{array}{cccc} 0 & \frac{v_{x}}{L(1+K_{us}v_{x}^{2})} & 0 & 0 \end{array}\right]}^{T}$$
  with the desired yaw rate as described in [25].
- **Q** is the positive semi-definite state weighting matrix:
  $$Q=\left[\begin{array}{cccc} 1e^{4} & 0 & 0 & 0\\ 0 & 1e^{4} & 0 & 0\\ 0 & 0 & 1e^{4} & 0\\ 0 & 0 & 0 & 1e^{4} \end{array}\right]$$
- **R** is the positive semi-definite control weighting matrix:
  $$R=\left[\begin{array}{cc} 1e^{-2} & 0\\ 0 & 1e^{-2} \end{array}\right]$$

The Hamiltonian function to solve the LQR problem is as follows [24]:

(24) $$H_{k}=\frac{1}{2}\left[\left(u_{c,k}^{T}{\mathrm{Ru}}_{c,k}\right)+{\left(x_{d,k}-x_{k}\right)}^{T}Q\left(x_{d,k}-x_{k}\right)\right]+P_{k}^{T}\left(A_{dc}x_{k}+C_{dc}u_{c,k}+B_{dc}v_{k}\right)$$

where:

- **P**${}_{k}$ is the Lagrange multipliers vector seen in Equation (25):
  (25) $$P_{k}=\left[P_{1},P_{2},P_{3},P_{3},P_{4},P_{5}\right]$$

In addition, the following equations must be satisfied as necessary conditions [24]:

(26) $$x_{c,k+1}=\left(\frac{\partial H_{k}}{\partial P_{k}}\right)=\left(A_{dc}x_{c,k}+C_{dc}u_{c,k}+B_{dc}v_{k}\right)$$

(27) $$P_{k}=-\left(\frac{\partial H_{k}}{\partial x_{k}}\right)=Q\left(x_{d,k}-x_{k}\right)-P_{k}^{T}A_{dc}=Q\left(x_{d,k}-x_{c,k}\right)-A_{dc}^{T}P_{k}$$

(28) $$0=\left(\frac{\partial H_{k}}{\partial u_{c,k}}\right)={\mathrm{Ru}}_{c,k}+P_{k}^{T}C_{dc}={\mathrm{Ru}}_{c,k}+C_{dc}^{T}P_{k}$$

Given that **P**${}_{k}$ can be written as:

(29) $$P_{k}={\mathrm{Kx}}_{c,k}+S$$

It can be obtained with Equations (27) and (29):

(30) $${\mathrm{Kx}}_{c,k+1}+S=Q\left(x_{d,k}-x_{c,k}\right)-A_{dc}^{T}P_{k}$$

Operating and substituting equivalent terms, Equation (30) can be written as:

(31) $$K\left(A_{dc}x_{c,k}-C_{dc}R^{-1}C_{dc}^{T}\left({\mathrm{Kx}}_{c,k}+S\right)+B_{dc}v_{k}\right)+S=Q\left(x_{d,k}-x_{c,k}\right)-A_{dc}^{T}\left({\mathrm{Kx}}_{c,k}+S\right)$$

**x**${}_{c,k}$ dependent terms can be separated from non-dependent terms obtaining:

(32) $$\left\{\begin{array}{c} K\left(A_{dc}x_{c,k}-C_{dc}R^{-1}C_{dc}^{T}{\mathrm{Kx}}_{c,k}\right)=-{\mathrm{Qx}}_{c,k}-A_{dc}^{T}{\mathrm{Kx}}_{c,k}\\ K\left(-C_{dc}R^{-1}C_{dc}^{T}S+B_{dc}v_{k}\right)+S={\mathrm{Qx}}_{d,k}-A_{dc}^{T}S \end{array}\right.$$

The gain matrix **K** for the controller was calculated according to [24]. δ and $\phi_{r}$ were considered as disturbances and the control variables determined by means of:

(33) $${U=-R}^{-1}B_{dc}^{T}(Kx_{c,k}+S)$$

where:

- **S** is a 4 × 4 matrix calculated solving the second Equation in (32):
  (34) $$S=-{\left[A_{dc}^{T}{-\mathrm{KB}}_{dc}R^{-1}B_{dc}^{T}\right]}^{-1}\left[{\mathrm{KB}}_{dc}u_{c,k}{-\mathrm{Qx}}_{d,k}\right]$$

Finally, the discrete Riccati equation (first Equation in (32)) was solved to obtain the value of **K** for every sample time.

## 5. Architecture of the Controller

To check the effectiveness of the LQR controller with road bank estimation, a series of tests was performed through the TruckSim software in combination with MATLAB-Simulink. An experimentally-validated vehicle model of a Mercedes Benz Sprinter van was used to carry out different tests in different environments [26]. The real vehicle was equipped with a vbox 3i dual antenna data logger, a steering angle sensor (MSW 250 Nm from Kistler (Winterthur, Switzerland)), an IMU (Inertial Measurement Unit) sensor and two GPS antennas from Raceologic (Buckingham, UK). The IMU was located in the floor of the van, near its center of gravity, while the two antennas were placed on the roof of the vehicle, forming 90${}^{{}^{\circ}}$ with respect to the traveling direction, to accurately measure the sideslip and roll angle.

**Figure 3:**
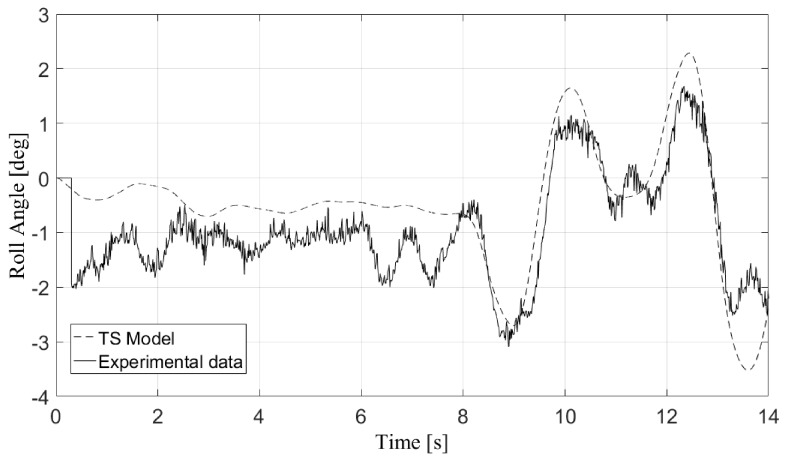
Validation of the vehicle simulation model for a double change lane maneuver.

**Figure 4:**
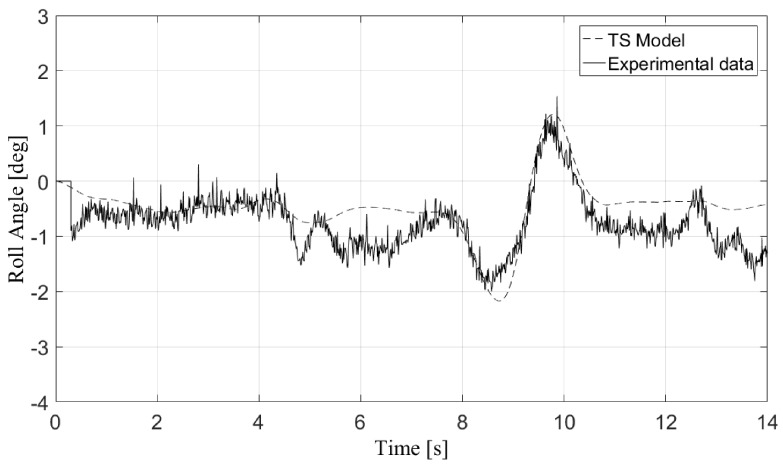
Validation of the vehicle simulation model for a change lane maneuver.

Figure 3 and Figure 4 depict the validation results for the roll angle of the vehicle in two of the maneuvers performed with the real vehicle: a double change lane and a simple change lane. TruckSim allowed these observable states to be exported to the controller, which could be measured with sensors in the real vehicle. During the vehicle model validation, the only inputs considered were the steering angle and the vehicle speed directly obtained from the sensors. Some perturbations such as road irregularities were not taken into account, which meant that the vehicle model did not totally capture the behavior of the real vehicle. Nevertheless, these errors showed that the vehicle model adequately represented the behavior of the real vehicle [26].

**Figure 5:**
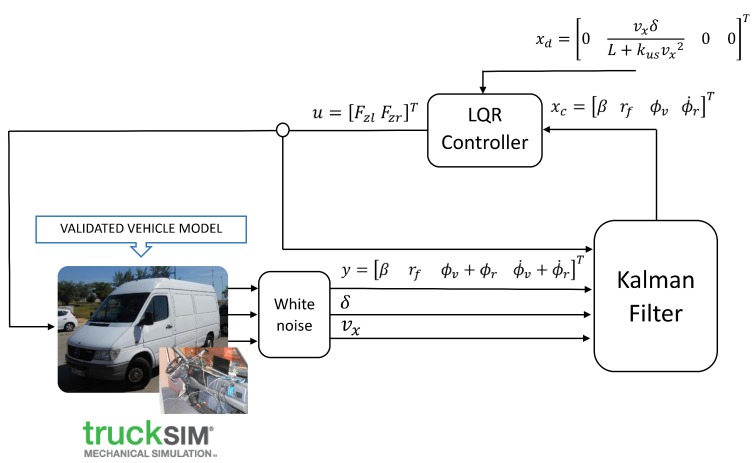
Controller structure.

The structure of the proposed controller is shown in Figure 5. Vector $y_{\mathrm{km}}$ (see Section 3) was obtained directly from the vehicle model, as well as the instantaneous velocity of the vehicle. The values for the vertical forces exerted by the active suspension system were looped back from the output of the LQR controller to the Kalman filter. As a flat vehicle model was used to perform control, a post-processing of the output signal was performed. By means of trial and error, it was set up to exert 70% of the calculated force on the rear axle (as rollover starts at this part of the vehicle) and 30% at the front of the vehicle. In this case, the estimated variables were the inputs of the LQR controller.

## 6. Results

To quantify the effectiveness of the controller, the load transfer coefficient, *R*, was calculated, which has been widely used in previous studies described in [27,28]. This coefficient indicates the proportion of the weight of the vehicle being withstood by each of the sides of the vehicle. It has been defined according to [27] as:

(35) $$R=\frac{\Delta F_{z}}{mg}$$

where m is the mass of the vehicle and $\Delta F_{z}$ is the load transfer. This coefficient has values in the range [−1, +1]. When these extreme values are achieved, it is considered that the vehicle’s roll stability is no longer guaranteed as the wheels on one side are starting to lift from the ground [28]. However, it was considered that the lift of one wheel did not necessarily mean rollover. The maximum values for the normalized load transfer coefficient, *R*, obtained for each simulation test are given in Table 1. These values were obtained solving Equation (35), where $\Delta F_{z}$ was calculated from vertical load on each tire with the TruckSim software.

In addition, the normal error, *E*${}_{k}$, for roll angle, yaw rate and sideslip angle with respect to the desired one was calculated. To perform these calculations, Equations (36)–(38) were used [26]:

(36) $$E_{k}=\frac{\varepsilon_{k}}{\sigma_{k}}$$

where:

(37) $$\varepsilon_{k}=\sum_{k=0}^{N-1}(\lambda_{\exp,k}-\lambda_{d,k})$$

(38) $$\sigma_{k}=\sum_{k=0}^{N-1}(\lambda_{\exp,k}-u_{k})$$

and:

- $\varepsilon_{k}$ is the approximation error.
- $\sigma_{k}$ is the standard deviation.
- $\lambda_{\exp,k}$ represents the measured variable.
- $\lambda_{d,k}$ is the desired value for each variable.
- *u*${}_{k}$ is the average value for each variable

In this section, the results obtained for the load transfer coefficient (Equation (35)) are shown for three of the performed maneuvers, as well as the vehicle’s roll angle, sideslip angle and yaw rate. These three simulations were selected to show the effectiveness of the LQR controller when compared with a fuzzy logic controller and a vehicle equipped with passive suspension. The detail of the design of the fuzzy controller is given in [28].

Simulation conditions were chosen to grow in demand for the controller, so that it could be tested in a wide range of situations such as low speed actuation and high inertia maneuvers with great non-linearities.

### 6.1. Test 1: 180-Degree Steering Wheel Turn

**Figure 6:**
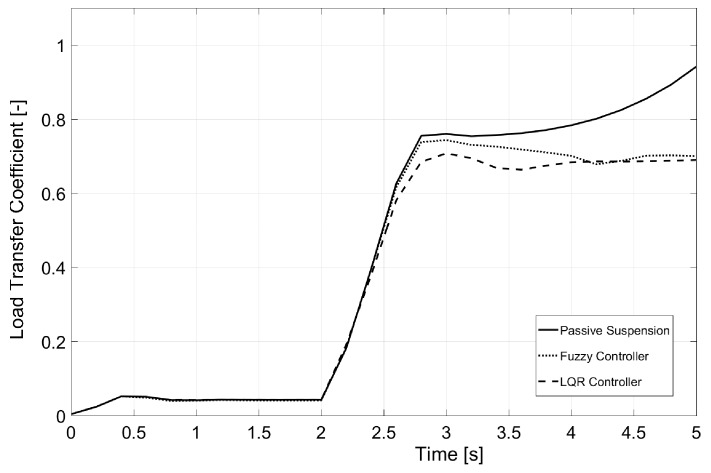
Load transfer coefficient for Test 1.

This first test was carried out in a flat environment that resembled an airfield. The van traveled with an initial velocity of 38 km/h, and on Second 2 of the simulation, the steering wheel turn was performed. As seen in Figure 6, the normalized transfer load coefficient reached a value of one using passive suspension, indicating that rollover was produced. At that moment, the simulation stopped. The response for two controllers (based on fuzzy and LQR) showed a reduction of about a 35% for the normalized load transfer coefficient. For both controllers, the vehicle did not rollover. The maximum value for the load transfer coefficient was smaller for the LQR controller than for the fuzzy controller.

In Table 2, it was observed that the normal error (calculated using Equation (36)) with respect to the desired vehicle roll (which was zero for every instance) was reduced up to 1.14 with the proposed system.

**Figure 7:**
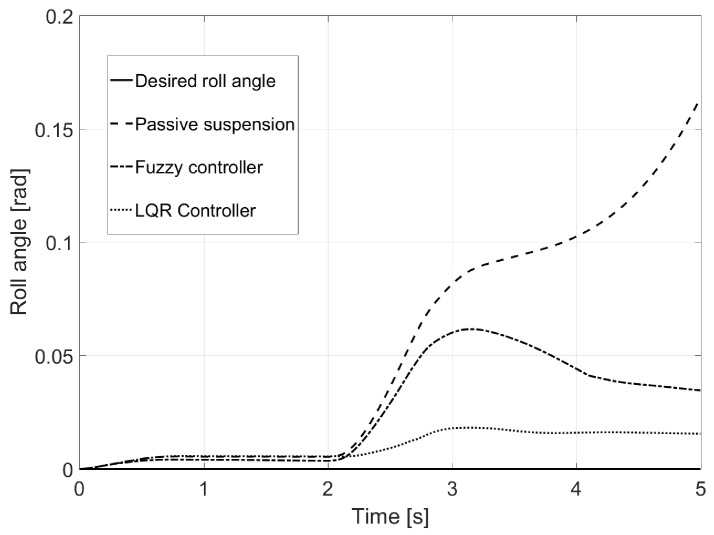
Roll angle for Test 1.

In Figure 7, the reduction of the vehicle’s roll angle was observed, which in the case of the LQR controller is close to the desired one.

**Figure 8:**
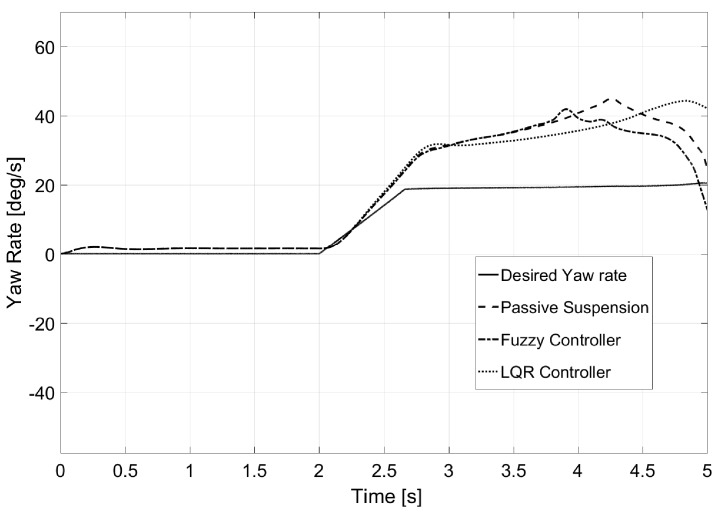
Yaw rate for Test 1.

Regarding yaw rate (see Figure 8), it was observed that both controllers prevented this value from increasing as rollover was avoided. Furthermore, a sudden decrease of the value was observed when rollover was produced for both passive suspension and the fuzzy logic controller. Values for calculated normal error are also presented in Table 2.

**Figure 9:**
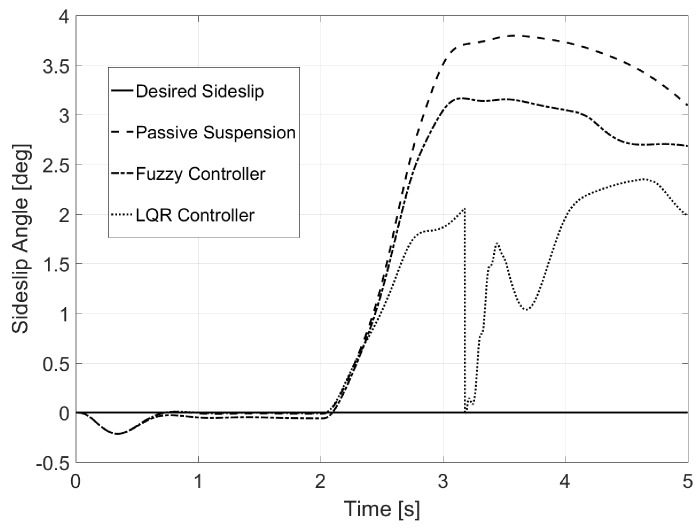
Sideslip for Test 1.

Figure 9 shows that the sideslip angle was reduced thanks to the LQR controller. The values for this variable were close to the desired one for every instance of the simulation, whereas the fuzzy controller was not able to show as good an improvement (see Table 2 for normal error values).

### 6.2. Test 2: Eight-Shaped Circuit

**Figure 10:**
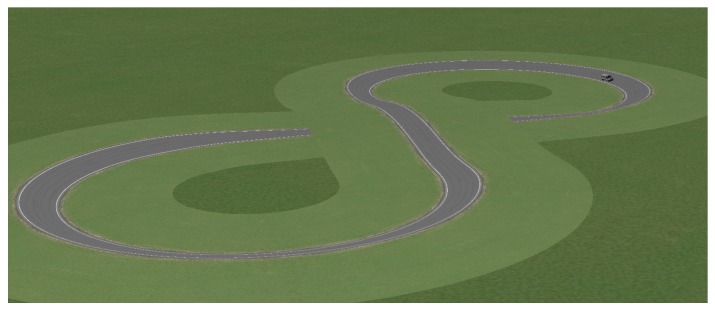
Global view for Test 2.

**Figure 11:**
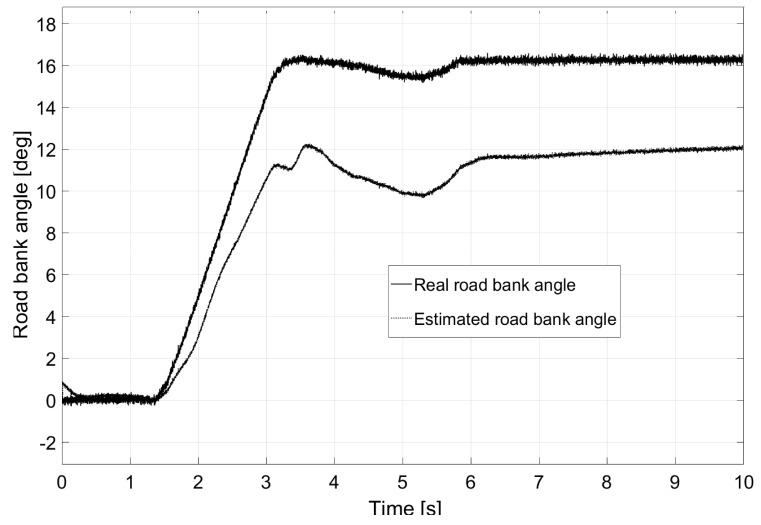
Bank angle estimation for Test 2.

The second test was performed in an “eight-shaped” closed circuit as shown in Figure 10. In this case, the vehicle travels at a constant speed of 80 km/h. Among the particularities of the test, it is worth mentioning that the road had a 30% bank on both turns (with a radius of 50 m) and a slope on the cross-section. This road bank variation is shown in Figure 11, as is the estimation performed by the Kalman filter.

**Figure 12:**
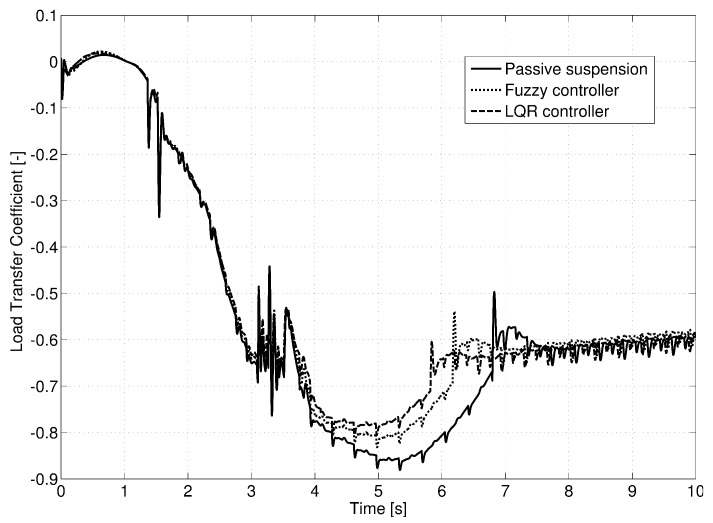
Load transfer coefficient for Test 2.

The evolution of the load transfer coefficient for this test is displayed in Figure 12. Ten seconds of the test are shown, which corresponded to entering into one of the turns going down the hill. In the most demanding instance, that is before the turning was stabilized, there was a great reduction in the lateral load transfer even though rollover did not take place for any of the vehicles. The LQR controller presented a quicker response for stabilizing the vehicle’s load transfer than the fuzzy logic controller; however, both of them avoided the lifting of the inner rear wheel of the van that was produced when passive suspension was equipped.

The normal error, $E_{k}$, was calculated for the vehicle’s roll angle, yaw rate and sideslip angle. The values (see Table 2) for both the fuzzy logic controller and the LQR controller were lower in this test than those corresponding to the vehicle with passive suspension.

**Figure 13:**
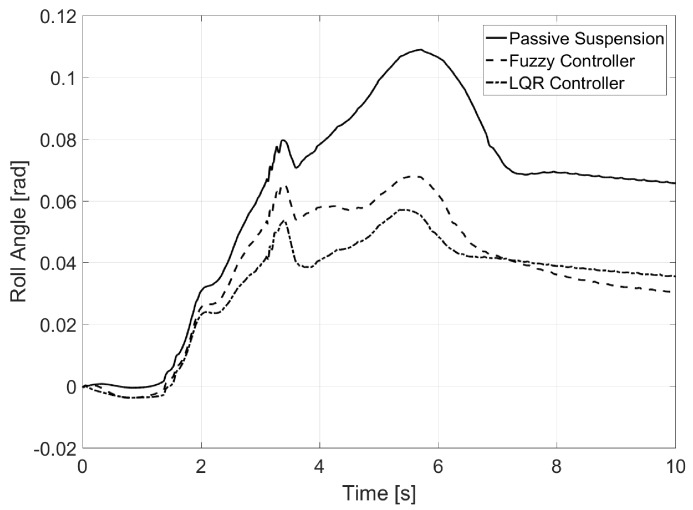
Roll angle for Test 2.

In Figure 13, roll angle was plotted for every instance. The LQR controller showed a reduction of almost 50% with respect to the vehicle equipped with passive suspension.

**Figure 14:**
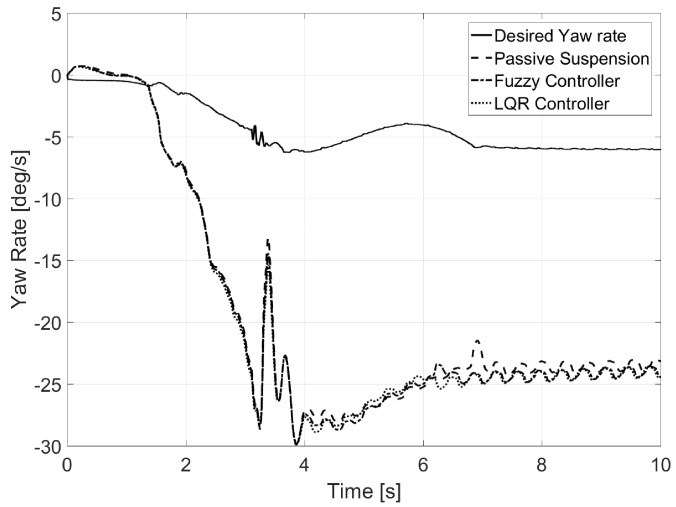
Yaw rate for Test 2.

Regarding yaw rate (see Figure 14), it was observed that the system did not negatively affect the lateral behavior of the vehicle, as the graphs were similar for the three shown vehicles.

**Figure 15:**
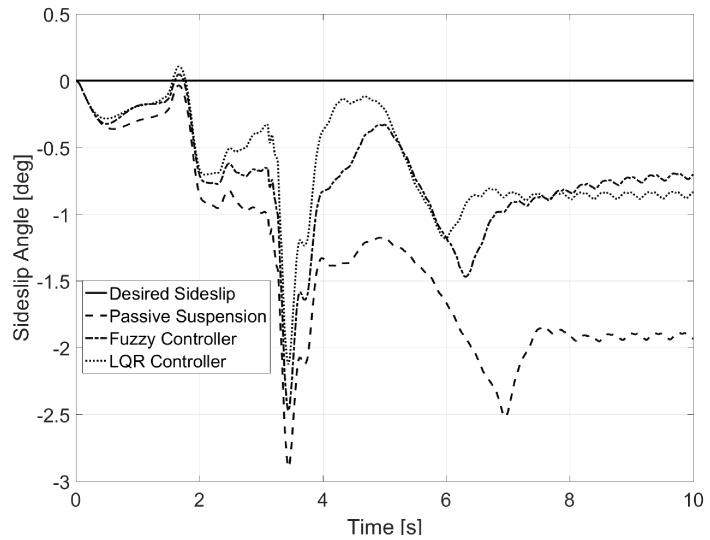
Sideslip for Test 2.

In the case of the aggressive bank, a great reduction of the sideslip angle was achieved, with almost 50% in some instances, as seen in Figure 15.

### 6.3. Test 3: Banked Road

**Figure 16:**
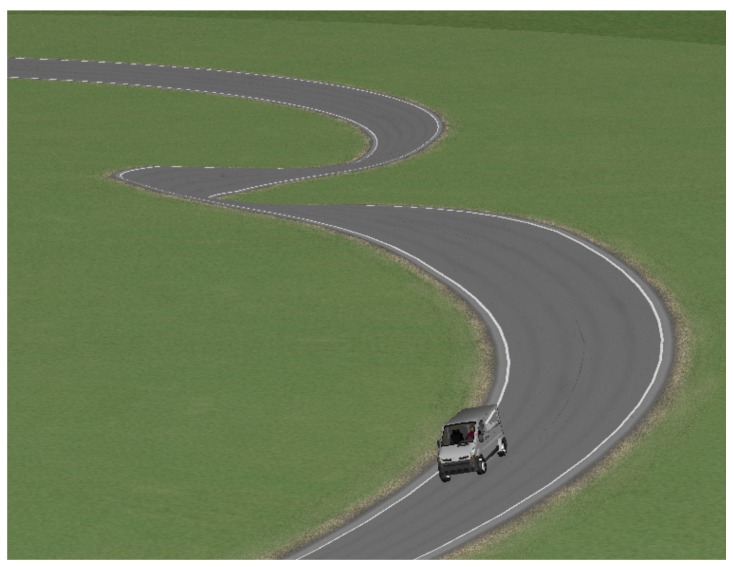
Global view for Test 3.

**Figure 17:**
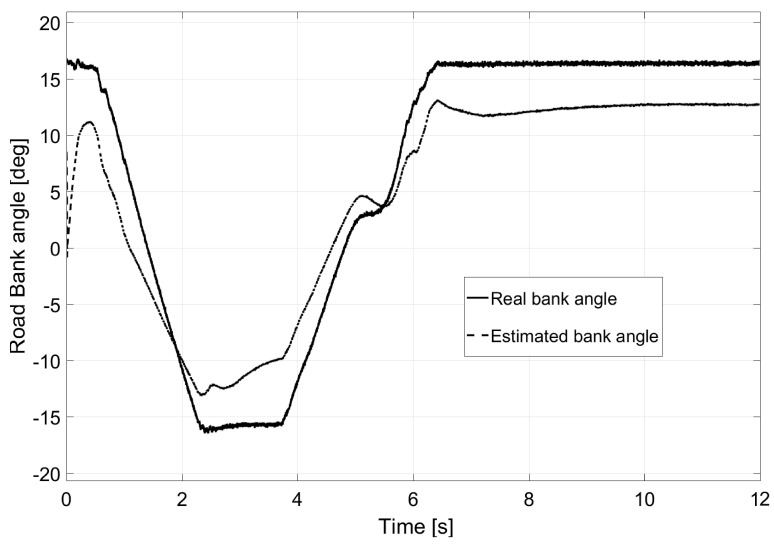
Bank angle estimation for Test 3.

**Figure 18:**
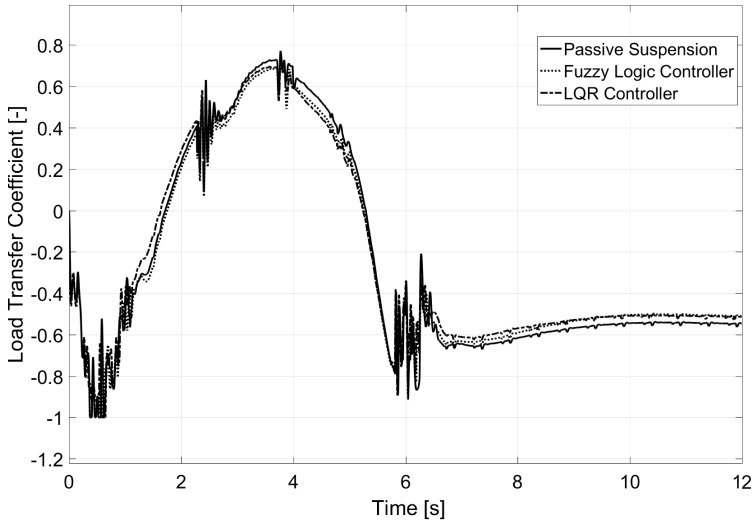
Load transfer coefficient for Test 3.

The last test presented was performed on the road shown in Figure 16. Consecutive turns with a radius of a 100 m were taken at a constant speed of 135 km/h. Given the characteristics of the chosen vehicle, its rollover and lateral control were considered non-achievable due to the demanding conditions of the test. The profile of the road’s bank appears in Figure 17 as does the estimation performed by the Kalman filter. However, as observed in Figure 18, a small reduction of load transfer was still achieved with the LQR controller.

The six first seconds of the simulation show the transition of the two turns. Compared to the previous environment described above, in this case, it is worth pointing out that a sudden change in the bank (from +30%–−30%) was produced. This caused a transient condition that could affect the control of the vehicle if the bank had not been taken into account. After this instance in the simulation, the van entered into the last turn, which was longer, and therefore, it had enough time to stabilize.

**Figure 19:**
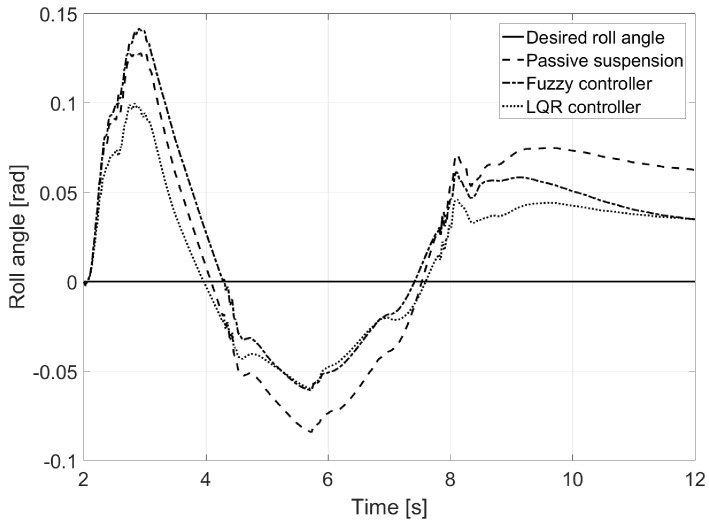
Roll angle for Test 3.

As seen in Table 2, the normal error for roll angle was reduced by up to 1.09. According to Figure 19, the LQR controller reduced the roll angle by 25% with respect to the one of the vehicles equipped with passive suspension, whereas the fuzzy controller showed an undesired peak of behavior in the first turn.

**Figure 20:**
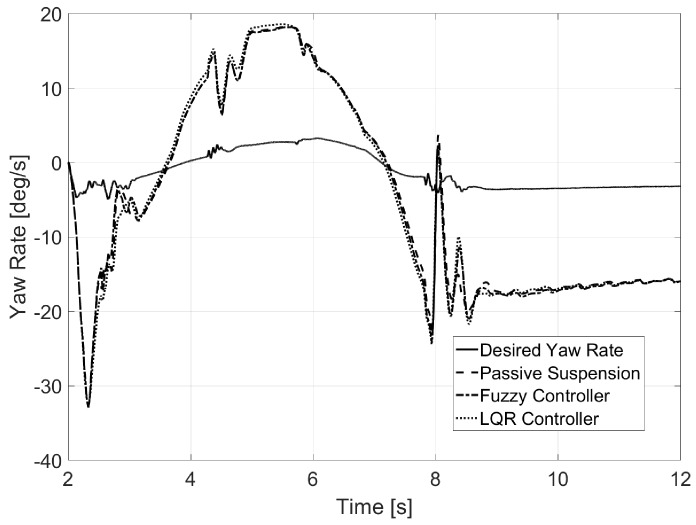
Yaw rate for Test 3.

The case for yaw rate (Figure 20) was similar to that shown in Test 2, as the active suspension system did not produce any undesired behavior at high speed.

**Figure 21:**
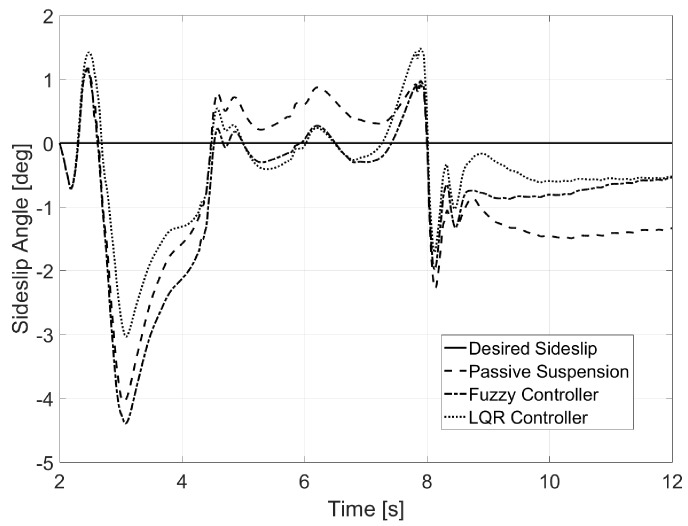
Sideslip for Test 3.

A reduction for the sideslip angle was also found in this last environment (see Figure 21), even though it was smaller than in the previous example, mainly because of the 135 km/h of traveling speed.

## 7. Conclusions

Our results showed big reductions of the load transfer and roll angle in almost every case studied (reducing the values up to 50%). The yaw rate was reduced in those simulations when performed at speeds up to 80 km/h, while sideslip angle improved for every case.

In addition, even though active suspension was used only for control, it was achieved with a controller that did not negatively affect lateral behavior in banked surfaces and improved it on flat environments (as heavy vehicles roll over before loosing lateral stability). Furthermore, this controller also prevented the oversteering effect, which can sometimes be produced by rollover controllers. The sideslip angle for every simulated case was shown to be smaller than the original one. 

## Figures and Tables

**Table 1:** Maximum load transfer coefficient, R.

	Test 1	Test 2	Test 3
Passive Suspension	1	0.85	0.75
Fuzzy Controller	0.75	0.8	0.65
LQR controller	**0.7**	**0.78**	**0.65**

**Table 2:** Normal error values, *E*${}_{k}$.

	Test 1	Test 2	Test 3
***Roll Angle,*** $\phi_{v}$			
Passive Suspension	1.38	4.59	1.35
Fuzzy Controller	1.21	3.20	1.38
LQR Controller	**1.14**	3.82	**1.09**
***Yaw Rate, $r_{v}$***			
Passive Suspension	3.04	0.60	0.63
Fuzzy Controller	3.04	0.60	0.64
LQR Controller	**2.91**	**0.60**	0.64
***Sideslip Angle, β***			
Passive Suspension	0.99	1.10	1.00
Fuzzy Controller	0.56	1.11	1.03
LQR Controller	**0.49**	**1.08**	**0.99**

## Nomenclature

***Symbol***	***Description***	***Value***	***Units***
*a*	Distance COG-front axle	1.51	(m)
*b*	Distance COG-rear axle	2.04	(m)
$b_{r}$	Roll damping coefficient	4.87 × 10${}^{4}$	(Nms/rad)
$C_{\alpha f}$	Front cornering stiffness	4.57 × 10${}^{4}$	(rad${}^{-1}$)
$C_{\alpha r}$	Rear cornering stiffness	8.14 × 10${}^{5}$	(rad${}^{-1}$)
$F_{zl}$	Vertical force on the left side	—	(N)
$F_{zr}$	Vertical force on the right side	—	(N)
*h*	Height From COG to the roll center	0.18	(m)
$I_{eq}$	Equivalent moment of inertia	1.38 × 10${}^{3}$	(kgm${}^{2}$)
$I_{x}$	Moment of inertia around the x axis	1.31 × 10${}^{3}$	(kgm${}^{2}$)
$I_{z}$	Moment of inertia around the z axis	4.33 × 10${}^{3}$	(kgm${}^{2}$)
$k_{r}$	Roll stiffness	3.71 × 10${}^{5}$	(Nm/rad)
*L*	Distance between the front and rear axle	3.55	(m)
*m*	Sprung mass	2150	(kg)
$P_{f}$	Roll rate of the vehicle frame	—	(rad/s)
$r_{f}$	Yaw rate of the vehicle frame	—	(rad/s)
$t_{r}$	Wheel track	1.63	(m)
$V_{x}$	Vehicle’s traveling speed		(m/s)
β	Sideslip angle	—	(rad)
δ	Steering wheel angle	—	(rad)
$\varepsilon_{r}$	Measurement error for yaw rate	—	(rad/s)
$\phi_{r}$	Road bank angle	—	(rad)
$\phi_{v}$	Vehicle roll angle	—	(rad)
